# Step-by-Step Guide to the Punch Removal Technique for Dermal Piercing: A Surgical Pearl

**DOI:** 10.7759/cureus.43516

**Published:** 2023-08-15

**Authors:** Adam J Elder, Hany Deirawan, Taylor Adlam, Meena Moossavi

**Affiliations:** 1 Department of Medical Education, Wayne State University, Detroit, USA; 2 Department of Dermatology, Wayne State University, Detroit, USA

**Keywords:** wound healing, quality improvement technique, piercing removal, dermatology, dermal piercing, cutaneous damage

## Abstract

While dermal piercings have become increasingly popular, there is limited dermatologic literature detailing a standard removal technique. Dermal piercings are often removed in the emergency department using non-serrated hemostats and a rocking motion until the anchor can be pulled through the skin. Removal by these means may lead to unnecessary damage to the skin, infections, and scarring. This article describes a straightforward technique for extracting dermal piercings that does not require the patient to know the size or type of dermal anchor. A detailed description, with corresponding images, is provided as a step-by-step guide on implementing a punch removal technique for dermal piercings. Dermatologists can implement this technique to remove piercings without knowing the underlying anchor type. This punch removal technique offers a solution for removing a variety of dermal piercings and subsequent scar tissue while minimizing scar formation and leaving patients with more cosmetically appealing skin.

## Introduction

Dermal piercings, also known as microdermal or single-point piercings, lie flat against the skin's surface. Unlike conventional piercings, dermal piercings do not have separate entry and exit points. Placement requires a large bore needle or punch and involves significant disruption of cutaneous integrity [1,2]. Dermal piercings can be placed anywhere in the body, typically without anesthetics. There are multiple types of dermal piercings. Usually, two pieces comprise a dermal piercing: the anchor and the internal post. [1] The anchor resides within the dermis, while the internal post protrudes externally but is attached to the anchor from one side. This allows for the jewelry to be connected externally. A "skin diver" is a dermal piercing placed in a single piece, often making it easier to remove. [1] Conventional piercings are more traditional, such as generic ear piercings, which can be taken out or replaced by the patient at any time. Knowledge of the underlying anchor type may simplify removal but is rarely available to dermatologists. Here, we describe a straightforward technique for extracting dermal piercings.

## Technical report

**Figure 1:**
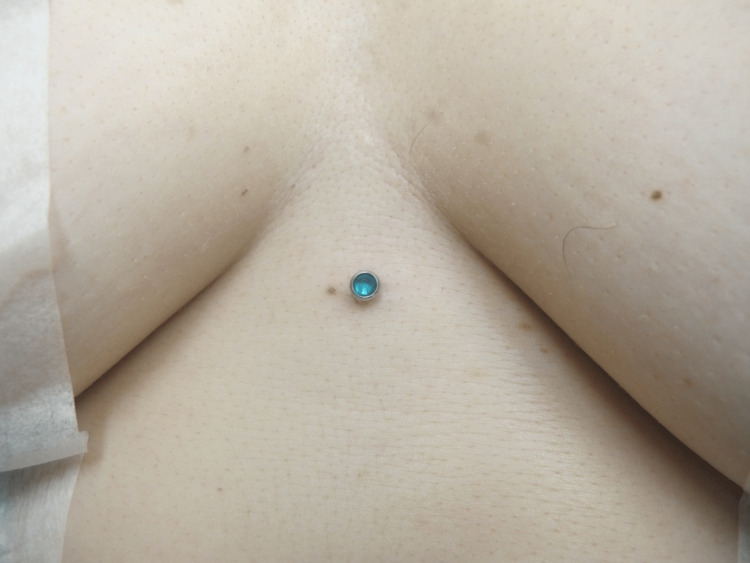
Central chest with dermal piercing prior to removal.

**Figure 2:**
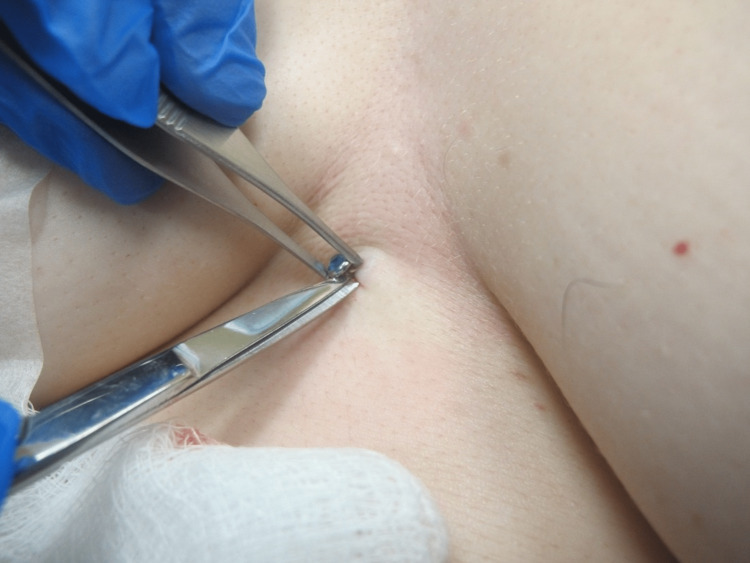
Dissection around post of dermal piercing with scissors after incision with a scalpel.

**Figure 3:**
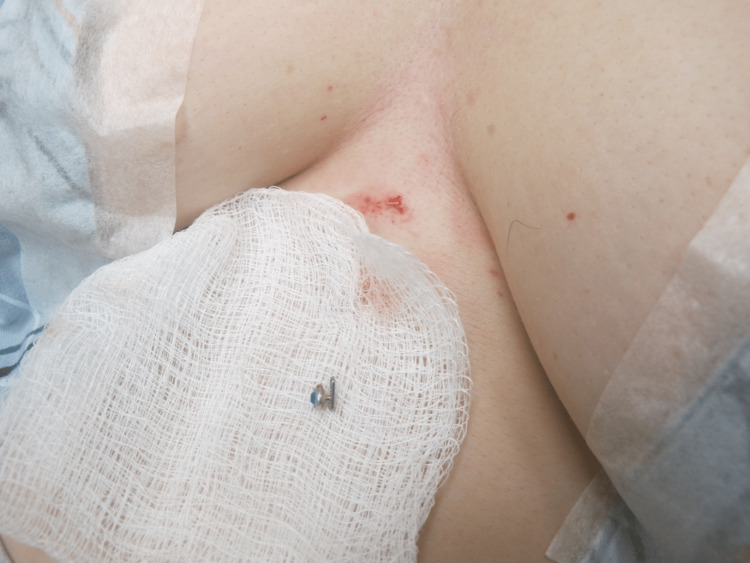
Dermal piercing after removal from skin and residual defect.

**Figure 4:**
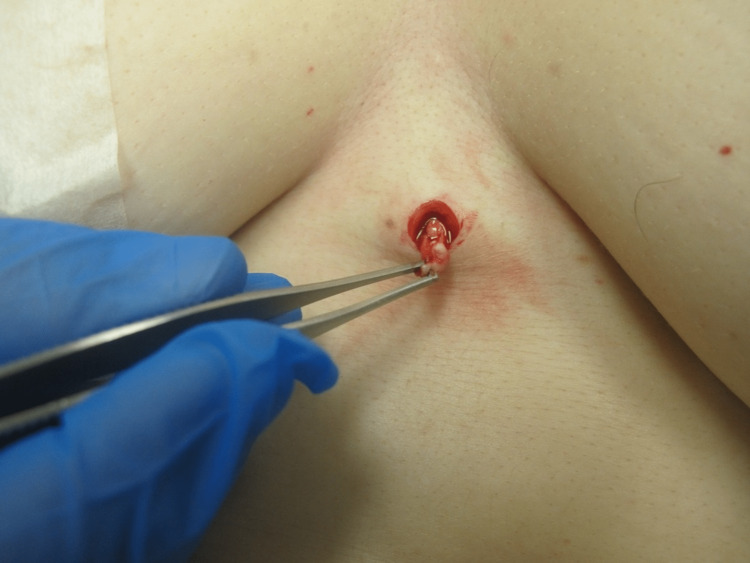
Irregular tissue removed by 5 mm punch tool after dermal piercing removal.

The most common complication of dermal piercings is a local infection, which the removal process can exacerbate. Preparing the site with a topical antiseptic and local anesthetic is essential [3,4]. Local anesthesia is usually achieved by infiltration of lidocaine 1% with 1:100,000 epinephrine injected around the piercing. Figure 1 demonstrates the dermal piercing prior to removal. The first incision aims to obtain exposure of the dermal piercings anchor, which usually coincides with the axis of maximum tension. This may be achieved by manipulating or "wiggling" the protruding jewelry attachment. Then, incise the skin using a scalpel or scissors near the base of the post and extend the incision parallel to the long axis of the piercing, if such axis is identified, or parallel to skin tension lines. Scissors can be used to gently dissect around the piercing, as illustrated in Figure 2. This approach may permit easy removal of the dermal anchor while minimizing the trauma of surrounding tissue regardless of anchor shape. Figure 3 demonstrates the defect after the removal of the piercing. Next, design an appropriate incision to excise the defect and scar or epithelized tract. A punch tool slightly larger than the piercing defect may facilitate the removal of all scar tissue in defects smaller than 8 mm. Figure 4 demonstrates the irregular tissue removed by the punch tool. Finally, depending on the size of the final defect and location, a simple or layered closure is executed to create a cosmetically favorable result. In this case, a deep dermal suture was placed using 4-0 Polyglactin 910 followed by two 4-0 nylon simple epidermal sutures. This technique offers a solution for removing a variety of dermal piercings and reducing complications resulting from residual scar tissue and epithelized tracts.

## Discussion

The basic structure of dermal piercing consists of a dermal anchor with an internally threaded post in the shape of an upside-down "T" [1]. The jewelry ornament attaches to the bar and can be changed. Dermal anchors often have holes in the base, allowing for tissue regrowth and added anchor strength. Another type of dermal piercing is the skin diver. Unlike a conventional dermal piercing, a skin diver combines the anchor, post, and jewelry into a single unit. Therefore, the jewelry attachment is not interchangeable. Skin divers may be easier to remove because their bases are often inverted cones without holes. Dermal anchors are offered in various shapes and sizes, frequently ranging between 3mm and 8mm. These materials are usually composed of surgical-grade stainless steel or titanium. Correct placement requires an incision with a scalpel or dermal punch to create space for the placement of the anchor, and the final result gives the appearance of beads flat against the skin's surface [1,5]. With any implantation of foreign material, there are risks of infection, immune reactions, and mechanical trauma, and many patients request the removal of the piercing.

While this class of piercings is increasingly popular, there is limited dermatologic literature to establish a standard technique for removal. Dermal piercings are often removed in the emergency department using non-serrated hemostats and a rocking motion until the anchor can be pulled through the skin [1,5]. Removal by these means may lead to unnecessary damage to the skin, scarring, persistent defect, or development of an inclusion cyst. The severity of complications varies with the piercing location, materials used in implantation and removal, and hygiene care regimens [6]. Winn et al. have proposed punch removal of dermal piercings without discussing the technique [1]. Here, we describe the punch removal technique for dermal piercings that does not require the patient to know the size or type of dermal anchor. The presumed benefits of such a technique include the excision of scar tissue from the piercing region and an esthetically more pleasant result. The clinical outcome proved the technique's effectiveness, displaying excellent scar cosmesis and patient satisfaction. The anticipated complications of our approach are similar to those related to epidermal inclusion cysts. In our described technique, we excise the piercing tract to minimize the odds of developing an epidermal inclusion cyst or a persistent dilated pore that may become secondarily infected.

## Conclusions

Dermal piercings are quite popular worldwide, as they serve as a method for self-expression and can even have cultural significance. Yet, dermal piercings' most commonly used removal methods pose multiple complications and lack cosmetic appeal to patients due to subsequent scar tissue formation. Our proposed punch removal technique for dermal piercings is associated with better wound healing with minimal scarring, limited risk for infection, and an overall decrease in cutaneous damage than the conventional methods of removing dermal piercings. This removal technique also offers a way to effectively remove dermal piercings without needing background information from the patient on the type of dermal anchor used. For larger piercings and skin inserts, an alternative approach for excision may be better suited, such as elliptical excision or the use of destructive methods, such as electrodesiccation, laser, or chemical processes, to ablate the epidermal lining of the piercing tract. Regardless of the technique employed, identifying and exploring the piercing track is essential for minimizing the excision and resulting scar. This article adds to the dermatologic literature by describing a step-by-step technique for removing dermal piercings.
